# Upright trunk and lateral or slight anterior rotation of the pelvis cause the highest proximal femur forces during sideways falls

**DOI:** 10.3389/fbioe.2022.1065548

**Published:** 2022-12-23

**Authors:** Svein Kleiven, Pooya Sahandifar

**Affiliations:** ^1^ Neuronic Engineering, KTH Royal Institute of Technology, Stockholm, Sweden; ^2^ Royal Institute of Technology, Stockholm, Sweden

**Keywords:** body posture, trunk angle, pelvis angle, femur forces, sideways falls

## Abstract

Whole-body models are historically developed for traffic injury prevention, and they are positioned accordingly in the standing or sitting configuration representing pedestrian or occupant postures. Those configurations are appropriate for vehicle accidents or pedestrian-vehicle accidents; however, they are uncommon body posture during a fall accident to the ground. This study aims to investigate the influence of trunk and pelvis angles on the proximal femur forces during sideways falls. For this purpose, a previously developed whole-body model was positioned into different fall configurations varying the trunk and pelvis angles. The trunk angle was varied in steps of 10° from 10 to 80°, and the pelvis rotation was changed every 5° from −20° (rotation toward posterior) to +20° (rotation toward anterior). The simulations were performed on a medium-size male (177 cm, 76 kg) and a small-size female (156 cm, 55 kg), representative for elderly men and women, respectively. The results demonstrated that the highest proximal femur force measured on the femoral head was reached when either male or female model had a 10-degree trunk angle and +10° anterior pelvis rotation.

## 1 Introduction

Human body models (HBMs) are practical tools for traffic safety studies (Iwamoto et al., 2002; Alvarez et al., 2014; Fahlstedt et al., 2016). The geometry and mechanical properties of the total human model for safety (THUMS) are based on a healthy mid-size young adult male (Iwamoto et al., 2002). HBM’s are generally positioned in a standing or seated position to model pedestrians or car occupants during different traffic accident scenarios. Another typical type of traffic accident is a single pedestrian fall (The Swedish Transport Administration, 2018), where the body configuration vary in the moments before a fall.

A fall can lead to different body configurations, and each of the body extremities can hit the ground first. However, it is demonstrated that sideways falls are the leading causes of hip fractures (Greenspan et al., 1998; Schwartz et al., 1998; Wei et al., 2001; Ensrud, 2013; Nasiri Sarvi and Luo, 2017; Galliker et al., 2022). Parkkari et al. (1999) found a majority of hip fractures occurring as a result of a fall and direct impact on the greater trochanter of the femur. Van Den Kroonenberg et al., 1996 conducted a laboratory study on sideways falls with six young, healthy adults. They were requested to fall onto the gymnastics mattress voluntarily and naturally. The mean trunk angle (the angle between the trunk and the vertical) was roughly 20°, and only two of the subjects could use their arm or hand to break the fall. Another more extensive study on 44 young individuals (31 were females) found an average trunk angle of 42° (Feldman and Robinovitch, 2007). Moreover, it was reported that in 98% of falls, the initial impact occurred to the upper extremities, followed by hip impacts. The pelvis angle varied in the range of -20 to 20°, with an average posterior rotation of 8° (Feldman and Robinovitch, 2007). Choi and Robinovitch (2018) explored the effect of the pelvis rotations toward posterior or anterior directions (pelvis angle) using a hip impactor simulator. They found that the 10-degree anterior pelvis rotation leads to the highest load on the femoral neck (Choi and Robinovitch, 2018). A recent subject-specific finite element simulation study (Galliker et al., 2022) indicated that the femoral neck reaction forces which was quantified at the acetabulum were higher in lateral or 15° anterior pelvis rotations compared to other anterior (30°) or posterior rotations (15, 30, 60, and 90°).

Finite element models can be used to better understand the effect of trunk and pelvis angles on the proximal femur forces measured on the femoral head during sideways falls. To the best of our knowledge, no previous study has investigated the effect of trunk and pelvis angles using whole body models. In the current study, a modified and validated THUMS whole-body model (Sahandifar and Kleiven, 2021) was positioned in the relevant sideways falling configurations for males and females. The corresponding proximal femur forces on the femoral head were evaluated to investigate the trunk and pelvis angles that lead to the highest proximal femur forces.

## 2 Method

**FIGURE 1:**
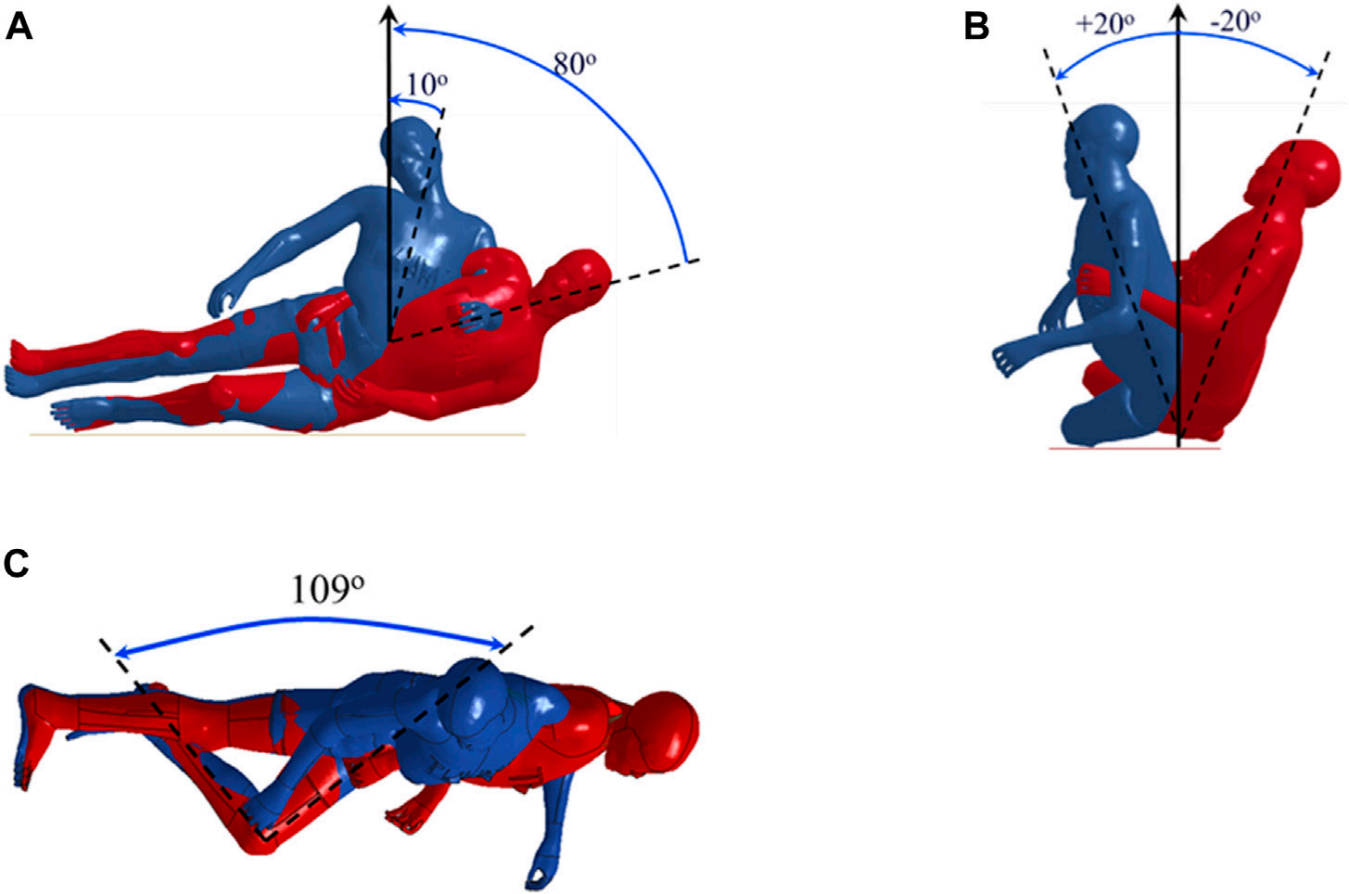
The range of the **(A)** trunk, **(B)** pelvis, and **(C)** knee angles during the sideways fall simulations.

The simulations were done using the modified THUMS v4.02 medium-sized male (Iwamoto et al., 2002) with a 177 cm height and 76 kg weight (Sahandifar and Kleiven, 2021), representative for elderly men (Kleiven, 2020; Fryar et al., 2021). Initially, the male model was homogenously scaled down to represent a small-size female with 156 cm height and a 56 kg weight (Schneider et al., 1983), which is close to the average height and weight reported for elderly women (Kleiven, 2020). Both models were validated against lateral impacts towards the pelvis for both external forces and internal forces of the femoral head in a previous study (Sahandifar and Kleiven, 2021). Next, the bones in the model were switched to rigid, during positioning, and the ipsilateral femur and lower leg were positioned to create a 109-degree knee-flexion (Van Den Kroonenberg et al., 1996; Fleps et al., 2018). A prescribed motion was assigned to the rigid bones to move to the desired position. After positioning the knee, different trunk and pelvis angles were positioned with the same method (Figure 1). Finally, the nodal positions were copied, and the bones returned to be deformable.

### 2.1 Sideways fall simulations

Sideways falls were simulated on a rigid ground with different trunk-angles and pelvis-angles (Figure 1). First, the trunk-angle was changed in steps of 10° from 10 to 80° compared with the vertical direction. According to the trunk-angle simulation results, the trunk-angle with the highest proximal femur force was chosen for simulating different pelvis-angles. The proximal femur forces were measured on the femoral head. The pelvis-angle was changed from −20° (rotation toward posterior) to +20° in steps of 5°. In all simulations, an initial velocity of 3 m/s was assigned to the positioned whole-body model as it is close to the average hip impact velocity reported in several previous studies (Feldman and Robinovitch, 2007; Nasiri Sarvi and Luo, 2017).

## 3 Results

**FIGURE 2:**
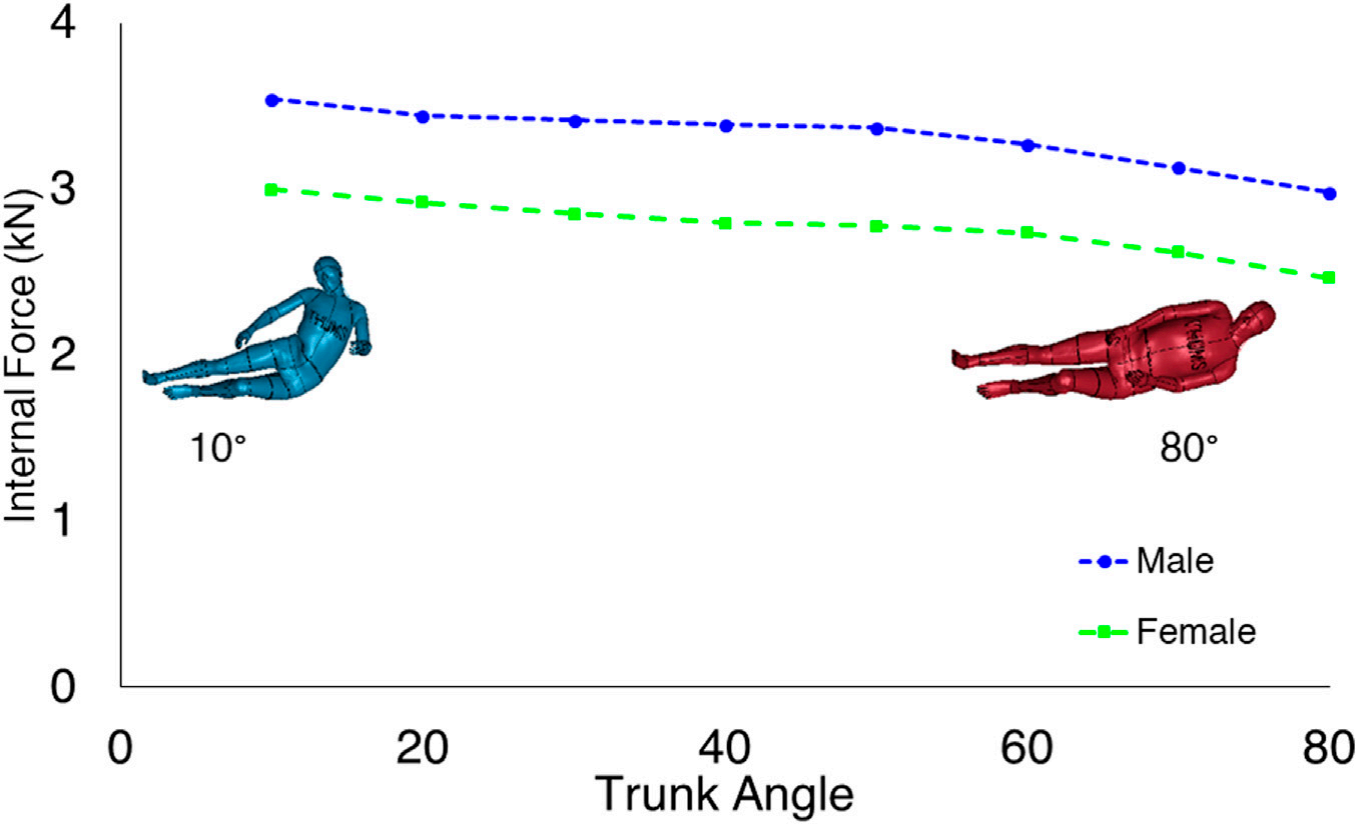
Comparison of the proximal femur forces in different trunk angles. The proximal femur forces decrease as the trunk angle reaches the horizontal body configuration.

The trunk-angle was changed from 10° to 80°, and the highest proximal femur forces were found for the most upright trunk position of 10° for both males and females (Figure 2). The female model experienced lower proximal femur forces (about 17 percent on average) than the male model at similar trunk angles.

**FIGURE 3:**
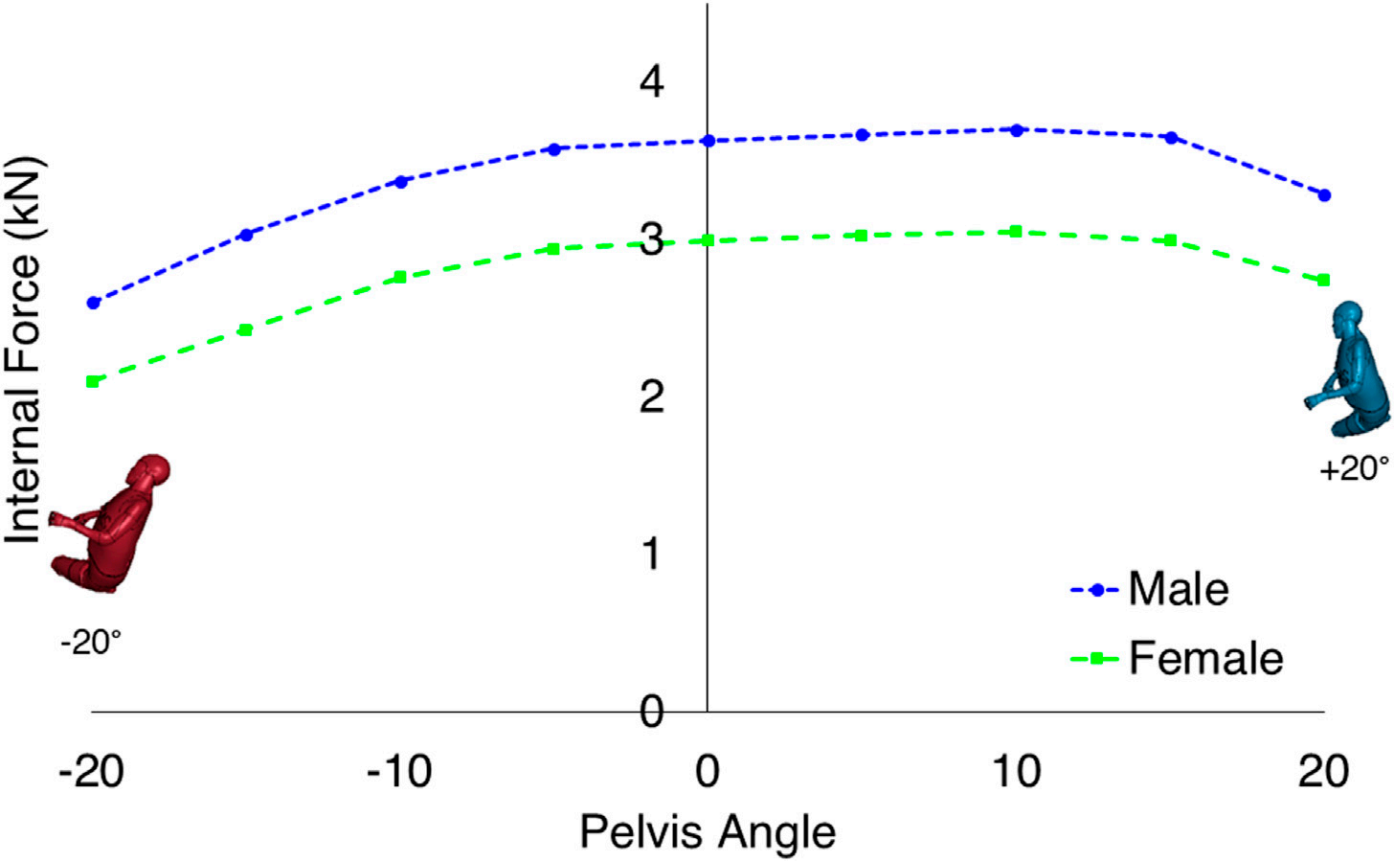
Comparison of the proximal femur forces in different pelvis angles when the trunk angle was fixed to 10°. The proximal femur force reaches the maximum value for both sexes at a pelvis angle of +10°.

The pelvis-angle was varied from 20-degree pelvis rotation towards posterior to 20-degree rotation towards anterior in steps of 5°. The highest proximal femur forces were found for pelvis angles of 0–15° anterior rotation for both males and females (Figure 3). The female model experienced lower proximal femur forces than the male model at similar body configurations.

## 4 Discussion

In the current study, the trunk and pelvis angles were varied to identify the body configuration leading to the highest proximal femur forces during sideways falls. The highest proximal femur force was found for the most upright trunk angle of 10° towards the vertical for both males and females; however, the highest proximal femur force due to changes in the pelvis angle occurred for the 10-degree anterior rotation. The proximal femur forces were, on average, 17 percent lower for the females than the males in each of the trunk and pelvis angles for the same hip impact velocity.

The results from the current study are supported by several previous studies (Choi and Robinovitch, 2018; Feldman and Robinovitch, 2007; van den Kroonenberg et al., 1995; Galliker et al., 2022). The proximal femur forces decrease as the trunk angle changes from 10 to 80°. The effective mass over the pelvis and the soft tissue thickness are the two factors contributing to the changes in the proximal femur forces due to trunk angle variations. Previous studies using spring-damper systems and linked rigid-body models have shown that the hip impact force increases as the trunk angle goes towards the vertical (Robinovitch et al., 1997; van den Kroonenberg et al., 1995). Simultaneously, the lateral flexion of the trunk could stretch the muscles and reduce the soft tissue thickness covering the greater trochanteric area. A previous study (Robinovitch et al., 1995) suggests a 1-mm increase in the soft tissue thickness can reduce the proximal femur forces up to 70 N. The finding of highest proximal femur forces is supported by the recent study by Galliker et al., 2022 who found highest femoral neck reaction forces for lateral or 15° anterior pelvis rotations for four subject specific lower body FE-models for lateral impacts. As the pelvis rotates toward the posterior, the thicker Gluteus muscles contribute more to the force attenuation of the fall. It consequently decreases the proximal femur forces. A positive pelvis-angle also changes the first point of impact from the hip toward the lateral and anterior parts of the hip having thinner soft tissues.

There are identifiable shortcomings with the current study. The trunk positioning of the whole-body model was limited to 10°. A smaller trunk angle than 10° would lead to distorted elements in the abdominal soft tissues due to folding of those layers. Moreover, the contralateral knee was not positioned with respect to the landing side knee. A recent study showed the landing side and contralateral knee configurations could affect the impact forces up to 60 percent in sideways fall (Lim and Choi, 2020). Only a single model for each sex is examined in this study. Although the two models are close to the average height and weight reported for elderly men and women, they do not account for normal population variations such as size, body mass, and soft tissue distribution. Another limitation is the homogenous scaling of a male model to obtain the female model. It was assumed that the differences between the female model and the male model was limited to the size differences, and other parameters such as geometry and mechanical properties were the same. However, it is indicated in previous studies such as Roberts et al., 2018 and Brinckmann et al., 1981 that the biomechanical differences such as bone structure and soft tissue composition cannot be ignored between sexes. While this limits the interpretation of the results when it comes to the female model, it should be noted that the female model correlates well when validated against lateral hip impacts for a small female PMHS with similar anthropometry (Sahandifar and Kleiven, 2021). Finally, it was assumed that the upper extremities were not involved in the impact, and the initial impact occurred to the hip. Van Den Kroonenberg et al., 1996 suggested that the subjects could not break the fall with their upper extremities, which is consistent with studies suggesting that elderly have difficulty breaking the fall (Parkkari et al., 1999), while Feldman and Robinovitch (2007) found that most of the volunteer impacts were initiated with one of the upper extremities and were followed by hip impact. Despite this discrepancy, the upper extremities could potentially absorb part of the impact forces and reduce the extent of impact forces applied to the hip. The involvement of the upper extremities could reduce the forces on the femoral neck and change the initial impact during sideways falls.

In conclusion, the femoral head undergoes the highest forces during the sideways falls when the model is positioned in a lateral or slight anterior pelvis rotation and an upright trunk angle of 10-degrees. The proximal femur force is found to be the highest at the same body posture for both the male and female models.

## Data Availability

The original contributions presented in the study are included in the article/supplementary material, further inquiries can be directed to the corresponding author.

## References

[B1] Alvarez V. S. Halldin P. Kleiven S. (2014). “The influence of neck muscle tonus and posture on brain tissue strain in pedestrian head impacts,” in SAE technical papers, SAE Int. 58, 63–101. 10.4271/2014-22-0003 26192950

[B2] Brinckmann P. Hoefert H. Jongen H. T. (1981). Sex differences in the skeletal geometry of the human pelvis and hip joint. J. Biomech. 14, 427–430. 10.1016/0021-9290(81)90060-9 7263735

[B3] Choi W. J. Robinovitch S. N. (2018). Effect of pelvis impact angle on stresses at the femoral neck during falls. J. Biomech. 74, 41–49. 10.1016/j.jbiomech.2018.04.015 29691053

[B4] Ensrud K. E. (2013). Epidemiology of fracture risk with advancing age. Journals Gerontol. - Ser. A Biol. Sci. Med. Sci. 68, 1236–1242. 10.1093/gerona/glt092 23833201

[B5] Fahlstedt M. Halldin P. Kleiven S. (2016). Comparison of multibody and finite element human body models in pedestrian accidents with the focus on head kinematics. Traffic Inj. Prev. 17, 320–327. 10.1080/15389588.2015.1067803 26218752

[B6] Feldman F. Robinovitch S. N. (2007). Reducing hip fracture risk during sideways falls: Evidence in young adults of the protective effects of impact to the hands and stepping. J. Biomech. 40, 2612–2618. 10.1016/j.jbiomech.2007.01.019 17395188

[B7] Fleps I. Vuille M. Melnyk A. Ferguson S. J. Guy P. Helgason B. (2018). A novel sideways fall simulator to study hip fractures *ex vivo* . PLoS One 13, e0201096. 10.1371/journal.pone.0201096 30040858 PMC6057661

[B8] Fryar C. D. Kruszon-Moran D. Gu Q. Carroll M. Ogden C. L. (2021). Mean body weight, height, waist circumference, and body mass index among children and adolescents: United States, 1999–2018. Hyattsville: National Health Statistics Reports. 10.15620/cdc:107559 34520341

[B9] Galliker E. S. Laing A. C. Ferguson S. J. Helgason B. Fleps I. (2022). The influence of fall direction and hip protector on fracture risk: FE model predictions driven by experimental data. Ann. Biomed. Eng. 50, 278–290. 10.1007/S10439-022-02917-0 35129719 PMC8847295

[B10] Greenspan S. L. Myers E. R. Kiel D. P. Parker R. A. Hayes W. C. Resnick N. M. (1998). Fall direction, bone mineral density, and function: Risk factors for hip fracture in frail nursing home elderly. Am. J. Med. 104, 539–545. 10.1016/S0002-9343(98)00115-6 9674716

[B11] Iwamoto M. Kisanuki Y. Watanabe I. Furusu K. Miki K. (2002). “Development of a finite element model of the total human model for safety (THUMS) and application to injury reconstruction,” in IRCOBI Conference Proceedings - International Research Council on the Biomechanics of Injury (Munich, Germany, 31–42.

[B12] Kleiven S. (2020). Hip fracture risk functions for elderly men and women in sideways falls. J. Biomech. 105, 109771. 10.1016/j.jbiomech.2020.109771 32423538

[B13] Lim K. T. Choi W. J. (2020). Effect of fall characteristics on the severity of hip impact during a fall on the ground from standing height. Osteoporos. Int. 31, 1713–1719. 10.1007/s00198-020-05432-x 32346772

[B14] Nasiri Sarvi M. Luo Y. (2017). Sideways fall-induced impact force and its effect on hip fracture risk: A review. Osteoporos. Int. 28, 2759–2780. 10.1007/s00198-017-4138-5 28730547

[B15] Parkkari J. Kannus P. Palvanen M. Natri A. Vainio J. Aho H. (1999). Majority of hip fractures occur as a result of a fall and impact on the greater trochanter of the femur: A prospective controlled hip fracture study with 206 consecutive patients. Calcif. Tissue Int. 65, 183–187. 10.1007/s002239900679 10441647

[B16] Roberts C. W. Forman J. L. Kerrigan J. R. (2018). Injury risk functions for 5 th percentile females: Ankle inversion and eversion. Conf. Proc. Int. Res. Counc. Biomech. Inj. IRCOBI 2018-Septe. 702–717.

[B17] Robinovitch S. N. Hayes W. C. Mcmahon T. A. (1997). Distribution of contact force during impact to the hip. Ann. Biomed. Eng. 25, 499–508. 10.1007/BF02684190 9146804

[B18] Robinovitch S. N. McMahon T. A. Hayes W. C. (1995). Force attenuation in trochanteric soft tissues during impact from a fall. J. Orthop. Res. 13, 956–962. 10.1002/jor.1100130621 8544034

[B19] Sahandifar P. Kleiven S. (2021). Influence of nonlinear soft tissue modeling on the external and internal forces during lateral hip impacts. J. Mech. Behav. Biomed. Mat. 124, 104743. 10.1016/j.jmbbm.2021.104743 34474319

[B20] Schneider L. Robbins D. Pflüg M. A. Snyder R. (1983). Development of anthropometrically based design specifications for an advanced adult anthropomorphic dummy family, volume 1. Final Rep.

[B21] Schwartz A. V. Kelsey J. L. Sidney S. Grisso J. A. (1998). Characteristics of falls and risk of hip fracture in elderly men. Osteoporos. Int. 8, 240–246. 10.1007/s001980050060 9797908

[B22] The Swedish Transport Administration (2018). Analysis of road safety trends 2017. Management by objectives for road safety work towards the 2020 interim targets. Analysis of road safety trends 2017. The Swedish Transport Administration. Available at: https://trafikverket.ineko.se/Files/sv-SE/57185/Ineko.Product.RelatedFiles/2019_035_analysis_of_road_safety_trends_2017_management_by_objectives_for_road_safety_work_towards_the_2020_interim_targets.pdf

[B23] van den Kroonenberg A. J. Hayes W. C. McMahon T. A. (1995). Dynamic models for sideways falls from standing height. J. Biomech. Eng. 117, 309–318. 10.1115/1.2794186 8618384

[B24] Van Den Kroonenberg A. J. Hayes W. C. McMahon T. A. (1996). Hip impact velocities and body configurations for voluntary falls from standing height. J. Biomech. 29, 807–811. 10.1016/0021-9290(95)00134-4 9147979

[B25] Wei T. S. Hu C. H. Wang S. H. Hwang K. L. (2001). Fall characterictics, functional mobility and bone mineral density as risk factors of hip fracture in the community-dwelling ambulatory elderly. Osteoporos. Int. 12, 1050–1055. 10.1007/PL00004184 11846332

